# *In vitro* anthelmintic effects of *Spigelia anthelmia* protein fractions against *Haemonchus contortus*

**DOI:** 10.1371/journal.pone.0189803

**Published:** 2017-12-15

**Authors:** Sandra Alves Araújo, Alexandra Martins dos Santos Soares, Carolina Rocha Silva, Eduardo Bezerra Almeida Júnior, Cláudia Quintino Rocha, André Teixeira da Silva Ferreira, Jonas Perales, Livio M. Costa-Júnior

**Affiliations:** 1 Laboratory of Plant Biochemistry, Chemical Engineering Course, Centre for Exact Sciences and Technology, Federal University of Maranhão, São Luíz MA, Brazil; 2 Laboratory of Parasite Control, Department of Pathology, Center for Biological and Health Sciences, Federal University of Maranhão, São Luíz MA, Brazil; 3 Laboratory of Botanical Studies, Department of Biology, Center for Biological and Health Sciences, Federal University of Maranhão, São Luís MA, Brazil; 4 Laboratory of Advanced Studies in Phytomedicines, Department of Chemistry, Centre for Exact Sciences and Technology, Federal University of Maranhão, São Luís MA, Brazil; 5 Laboratory of Toxinology, Oswaldo Cruz Foundation, Rio de Janeiro RJ, Brazil; Instituto Butantan, BRAZIL

## Abstract

Gastrointestinal nematodes are a significant concern for animal health and well-being, and anthelmintic treatment is mainly performed through the use of chemical products. However, bioactive compounds produced by plants have shown promise for development as novel anthelmintics. The aim of this study is to assess the anthelmintic activity of protein fractions from *Spigelia anthelmia* on the gastrointestinal nematode *Haemonchus contortus*. Plant parts were separated into leaves, stems and roots, washed with distilled water, freeze-dried and ground into a fine powder. Protein extraction was performed with sodium phosphate buffer (75 mM, pH 7.0). The extract was fractionated using ammonium sulfate (0–90%) and extensively dialyzed. The resulting fractions were named LPF (leaf protein fraction), SPF (stem protein fraction) and RPF (root protein fraction), and the protein contents and activities of the fractions were analyzed. *H*. *contortus* egg hatching (EHA), larval exsheathment inhibition (LEIA) and larval migration inhibition (LMIA) assays were performed. Proteomic analysis was conducted, and high-performance liquid chromatography (HPLC) chromatographic profiles of the fractions were established to identify proteins and possible secondary metabolites. *S*. *anthelmia* fractions inhibited *H*. *contortus* egg hatching, with LPF having the most potent effects (EC_50_ 0.17 mg mL^-1^). During LEIA, SPF presented greater efficiency than the other fractions (EC_50_ 0.25 mg mL^-1^). According to LMIA, the fractions from roots, stems and leaves also reduced the number of larvae, with EC_50_ values of 0.11, 0.14 and 0.21 mg mL^-1^, respectively. Protein analysis indicated the presence of plant defense proteins in the *S*. *anthelmia* fractions, including protease, protease inhibitor, chitinase and others. Conversely, secondary metabolites were absent in the *S*. *anthemia* fractions. These results suggest that *S*. *anthelmia* proteins are promising for the control of the gastrointestinal nematode *H*. *contortus*.

## Introduction

Gastrointestinal nematodes are a serious concern for animal health and well-being [1]. In addition, diseases caused by gastrointestinal parasites in small ruminants have resulted in significant animal husbandry losses both in Brazil and around the world [2,3]

Due to its widespread occurrence and high pathogenicity, *Haemonchus contortus* is the most important species among small ruminant nematodes [2,4]. Anthelmintic treatment of these parasites is currently performed mainly through the use of commercially available chemical products [5]. However, as a consequence of the high dosages applied and misuse of antiparasitic drugs, selection of resistant parasites has rendered these products ineffective [6]. Indeed, increased resistance has caused major economic impacts on livestock worldwide [3]. Alternatively, bioactive compounds produced by plants have shown promise for the development of novel anthelmintic products [7].

*Spigelia anthelmia* is an herbaceous species of the Loganiaceae family. Commonly known as "erva-lombrigueira", it is popular as a human medicine in Brazil based on its various properties, including action against gastrointestinal nematodes [8]. *In vitro* and *in vivo* studies have confirmed the efficacy of *S*. *anthelmia* in controlling *H*. *contortus* [9,10]; however, despite the great diversity of compounds present in plants, only secondary metabolites have been noted for being responsible for such biological action [11,12]. In addition to offering evidence for the anthelminthic activity of *S*. *anthelmia* extracts, identification of active protein compounds can improve extraction efficacy, thus reducing cost.

Although plant defense proteins have shown nematicidal properties, indicating their potential for developing new products to control *H*. *contortus* [13,14,15], few studies have been conducted on use against nematodes. Within this context, we report the anthelmintic activity of *S*. *anthelmia* protein fractions on eggs and infective larvae of the gastrointestinal nematode *H*. *contortus*.

## Materials and methods

### Plant material

*S*. *anthelmia* specimens were collected at Federal University of Maranhão in São Luís (2°33'14" S and 44°18'20" W), Maranhão State, Brazil, with accordance of responsible authority. The plant samples were sent to the Herbarium of Maranhão—MAR for botanical identification (voucher-5626). The plant material was washed thoroughly with distilled water, divided into leaves, stems and roots and immediately frozen for freeze-drying [16]. The plant material was ground into a fine powder and stored at -4°C for analysis.

### Extracts and protein fractions of *S*. *anthelmia*

Soluble proteins were extracted from the powdered plant samples with sodium phosphate buffer (75 mM, pH 7.0) containing 75 mM NaCl at a ratio of 1:20 (w/v) in the presence of 1% (w/v) polyvinylpolypyrrolidone (PVPP) to assist in the removal of phenolic compounds [17]. Leaf, stem and root extracts were obtained under moderate agitation at 4°C for 1 h. After extraction, the samples were centrifuged at 15,000 x g for 30 minutes at 4°C, and the supernatants were centrifuged under the same conditions. The resulting supernatants were dialyzed (*cut-off*: 14 kDa) and fractionated with ammonium sulfate at concentrations from 0–90% [18] which was gradually added in an ice bath under moderate agitation. The samples were then allowed to stand for 12 hours at 8°C and centrifuged at 4°C, 15,000 x g for 30 minutes. After this step, the supernatant was removed, and each pellet was resuspended in extraction buffer and subjected to exhaustive dialysis (*cut-off*: 14 kDa) against distilled water. Secondary metabolites and low-molecular-weight peptides were not retained in the protein solution. The protein fractions were named leaf protein fraction (LPF), stem protein fraction (SPF) and root protein fraction (RPF).

### Protein contents and protease, protease inhibitor, chitinase and lectin activity

The protein concentration was determined using a described method with bovine serum albumin (BSA) as the standard [19].

Proteolytic activity was measured using azocasein as a non-specific substrate [20]. One unit of activity (UA) was defined as the amount of enzyme at 0.01 mL^-1^ capable of increasing the absorbance at 420 nm within 60 min. Cysteine protease inhibitor activity was determined by measuring inhibition of papain activity using benzoyl-arginine-naphthylamide (BANA) as the substrate [21]. One unit of inhibitor activity (UI) was defined as the decrease in 0.01 absorbance units at 540 nm mL^-1^ min^-1^ compared with the control (papain activity in the absence of the inhibitor). Chitinase activity was determined using a colorimetric procedure that detects N-acetyl-D-glucosamine (NAG) produced by the combined hydrolytic action of chitinase and β-glucuronidase on the non-radioactive substrate known as colloidal chitin. The absorbance was measured at 585 nm, and chitinase activity was calculated as nanokatals (nkat) per milligram of protein [22]. Lectin activity was assessed using serial dilutions of samples with rabbit erythrocytes (2%) [23]. One hemagglutination unit (HU) was defined as the inverse of the dilution contained in each well. Specific hemagglutination activity was calculated as the titer ratio by determining the protein concentration in mg mL^-1^.

### Biological assays

#### Obtaining nematodes

*H*. *contortus* eggs and third-stage larvae (L_3_) were recovered from the feces of experimentally infected sheep. The experimental procedures were performed in accordance with the guidelines by the Animal Ethics Committee of the Federal University of Maranhão, and were approved by this committee under protocol number 23115018061/2011-11.

#### Egg hatching assay (EHA)

The ability of the samples to inhibit egg hatching was evaluated according to described methodology [24]. Each sample (100 μL) was added to a well of a 96-well sterile plate, and approximately 100 eggs were added. The samples were tested at pre-standardized protein concentrations (2.0, 1.0, 0.5, 0.25, 0.125 and 0.062 mg mL^-1^) in quadruplicate. For the control, eggs were incubated with the same buffer used to dissolve the extracts (10 mM sodium potassium phosphate buffer, pH 7.2, containing 125 mM NaCl). All plates were incubated for 48 h at 27°C and ≥80% relative humidity (RH). After this period, 50 μL of lugol was added to each well, and the eggs and hatched larvae were quantified to calculate the percent inhibition of larval hatchability.

#### Larval exsheathment inhibition assay (LEIA)

Larval exsheathment inhibition assay (LEIA) was performed according to a previously described method [25]. Viable *H*. *contortus* larvae (L_3_) were immersed in different concentrations of pre-standardized protein (1.2, 0.6, 0.3, 0.15 and 0.075 mg mL^-1^) and incubated for 3 h at 27°C and ≥80% RH. The larvae were then washed for three minutes in distilled water and centrifuged at 2,540 x g; this process was repeated twice. Immediately after washing, 1,000 larvae/tube were subjected to an artificial exsheathment process by contact with sodium hypochlorite (2.0%, w/v). Four replicates were performed for each treatment. The same buffer used to dissolve the extracts (10 mM sodium potassium phosphate buffer, pH 7.2, containing 125 mM NaCl) was used for the control. The kinetics of larval exsheathment in the different treatments was monitored at 0-, 20-, 40- and 60-min intervals by microscopic observation at a 100× magnification, and the percentages of exsheathed larvae were recorded.

#### Larval migration inhibition assay (LMIA)

Evaluation of larval migration inhibition was achieved using described methodology [26]. Initially, *H*. *contortus* larvae (L_3_) were subjected to the exsheathment process by contact with sodium hypochlorite solution (2%). After sieving, the larvae were centrifuged in distilled water for 5 minutes at 407 x g, and the supernatant was removed. The larvae were resuspended in distilled water and centrifuged. This process was repeated twice, until all the sodium hypochlorite solution was removed. The larvae were collected at approximately 1,000 larvae mL^-1^. A protein sample (1,000 μL) was added at different protein concentrations (1.0, 0.5, 0.25, 0.125, 0.06 and 0.03 mg mL^-1^) to 100 μL of the larval suspension containing 100 larvae. The suspensions were incubated for 2 h at 27°C and ≥80% RH. The tubes were then centrifuged at 1,500 x g for 10 minutes, and the supernatant was removed, reducing the volume by approximately 300 μL.

A 200-μL aliquot of the samples at the above concentrations were added to the wells of culture plates, and an apparatus containing 25-μm sieves was submerged into each well. A 50-μL volume of larval suspension was added to the corresponding apparatus followed by incubation for 2 h at 27°C and ≥80% RH. After this time, each apparatus was carefully removed and washed to withdraw the larvae contained in the mesh. A 10-μl volume of lugol was added to each well of the culture dish, and the larvae in each well and each filter were counted using an inverted microscope at a magnification of 100x. The assay was performed in quadruplicate. As a control, 10 mM sodium potassium phosphate buffer at pH 7.2 containing 125 mM NaCl was used.

### Proteomic analysis (liquid chromatography-electrospray ionization-tandem mass spectrometry, LC-ESI-MS/MS)

Aliquots of the *S*. *anthelmia* fractions were enzymatically digested with trypsin. Fifty micrograms of proteins were reduced with dithiothreitol and alkylated with iodoacetamide and then incubated for 16 h with 1 microgram of trypsin, in the proportion of 1/50 (w / w) enzyme / substrate. Each sample was analyzed in technical triplicate using a 24-cm reverse-phase (RP) column coupled to an LTQ-Orbitrap XL mass spectrometer (Thermo Scientific, San Jose, CA, USA) using the nLC-Easy II system (Thermo Scientific). The samples were applied to the pre-equilibrated column in 0.1% (v/v) formic acid (eluent A), and peptides were eluted using a gradient of 2 to 40% acetonitrile containing 0.1% formic acid (v/v) (eluent B). The peptides were sequentially subjected to collision-induced dissociation fragmentation (Collision Induced Dissociation, CID) and then analyzed by Linear Trap Quadrupole (LTQ) in MS/MS mode. The peptide mass profiles were evaluated using Peaks Studio 8.0 build 20160908 [27]. Searches were performed using the Uniprot viridiplantae database (Searched Entry: 4,822,572). The search parameters for monoisotopic peptide masses allowed two missed enzymatic cleavages and accepted cysteine residue carbamidomethylation with fixed modification and methionine oxidation as variables, fragment mass error tolerance of 0.6 Da. The false discovery rate (FDR) values at protein levels was ≤1%. To avoid redundancy, each confident protein identification involved at least one unique peptide. Redundancy identified in the resulting list (proteins with the same values for -10lgp; coverage, #peptides; #unique, PTM; Avg Mass) was manually removed.

### Qualitative high-performance liquid chromatography analysis (HPLC)

A Shimadzu model HPLC system (Shimadzu Corp., Kyoto, Japan) was used, consisting of a solvent delivery module with a double-plunger reciprocating pump and a UV-VIS detector (SPA-10A); a Luna 5 μm C18 100 A (250.0 μm x 4.6 μm) column was applied. The elution solvents A and B were 2% acetic acid in water and methanol, respectively. The sample injection volume was 20 μL. Elution was performed according to the following gradient: 95% A/5% B as the initial elution over 30 minutes at a flow rate of 1 mL min^-1^. The run time was 35 min, and the column temperature was 20°C. Data were collected and processed using LC Solution software (Shimadzu).

### Statistical analysis

Biochemical data were obtained in triplicate, and the results are expressed as means ± standard deviations. The probit method was used to calculate the effective concentration (EC) of each sample in terms of biological activity (against eggs and larvae). One protein fraction was considered to be significantly (P<0.05) more (or less) efficient than another if there was no overlap between the 95% confidence intervals of the EC_50_ values [28]. All analyses were performed using GraphPad Prism software v. 6.0 [29].

## Results

### Fraction protein content and activity of plant defense-related proteins

After total extracts were obtained from the leaves, stems and roots of *S*. *anthelmia* and fractionated with ammonium sulfate, the protein contents of each sample were determined. The total protein contents of LPF, SPF and RPF were 51.71, 9.19 and 8.28 mg, respectively.

Proteolytic activity was detected in all fractions, with higher activity in LPF and RPF (569.13 ± 23.1 and 219.42 ± 10.2 AU, respectively). Cysteine protease inhibitor activity was detected in LPF (111.31 ± 17.3 IU), SPF (80.04 ± 6.0 IU) and RPF (117.21 ± 15.0 IU). In contrast, chitinase activity was low, and lectin activity was not detected (Table 1).

**Table 1:** Proteolytic activity (AU), cysteine protease inhibitory activity (IU), chitinase activity (nkat), and lectin activity (HU) of protein fractions obtained from *Spigelia anthelmia*.

Protein activity	Fractions		
	LPF	SPF	RPF
Protease (AU)	569.13 ± 23.1	36.45 ± 0.9	219.42 ± 10.2
Cysteine protease inhibitor (IU)	111.31 ± 17.3	80.04 ± 6.0	117.21 ± 15.0
Chitinase (nkat)	0.01 ± 0.0	0.01 ± 0.0	0.07 ± 0.0
Lectin (HU)	-	-	-

LPF: leaf protein fraction; SPF: stem protein fraction; RPF: root protein fraction. Values are means ± standard deviation (SD) in triplicate.—indicates not detected.

### Identification of proteins in fractions

Mass spectrometry of the *S*. *anthelmia* fractions revealed 1,796, 2,516 and 1,829 proteins in leaves, stems and roots, respectively (S1 Fig). A total of 142 proteins were common to all fractions (S2 Fig), including ATP sintase (A0A1B4ZAZ5), fructokinase (Q7XJ81), ribosomal protein (A0A118K3B3), glutathione reductase (W6EAU1), calmodulin (Q3LRX2), protease (M1C4F2) and chitinase (different access). Ribonuclease was identified in LPF. A protease inhibitor was identified in SPF only. All fractions were free of secondary metabolic compounds, as confirmed by HPLC-UV (data not shown).

### *In vitro* tests against *Haemonchus contortus*

All fractions presented efficacy in terms of inhibiting *H*. *contortus* egg hatchability, with EC_50_ values of 0.17, 0.65 and 0.79 mg mL^-1^ for LPF, SPF and RPF, respectively (Table 2, S3 Fig).

**Table 2:** Half maximal effective concentration (mg mL^-1^; EC_50_) obtained for protein fractions of *Spigelia anthelmia* against *Haemonchus contortus* eggs and larvae.

Extracts	EC_50_ mg mL^-1^ (CI 95%)		
	EHA	LEIA	LMIA
**LPF**	0.17 (0.16–0.18)^a^	0.47 (0.37–0.59)^a^	0.21 (0.19–0.25)^b^
**SPF**	0.65 (0.60–0.70)^b^	> 1.20^c^	0.14 (0.11–0.17)^a^
**RPF**	0.79 (0.74–0.84)^c^	0.78 (0.73–0.83)^b^	0.11 (0.08–0.14)^a^

LPF: leaf protein fraction; SPF: stem protein fraction; RPF: root protein fraction. EHA: egg hatch assay; LEIA: larval exsheathment inhibition assay; LMIA: larval migration inhibition assay. 95% CI = 95% confidence interval. Different letters represent significant differences between treatments (P>0.05).

Activity was observed for LPF (EC_50_ 0.47 mg mL^-1^) and RPF (EC_50_ 0.78 mg mL^-1^) in LEIA (Table 2), whereas SPF at 1.2 mg mL^-1^ inhibited only 7.9% of larval exsheathment after 60 minutes of incubation (S3 Fig). The fractions also inhibited larval migration, with EC_50_ values varying from 0.11 to 0.21 mg mL^-1^ (Table 2).

## Discussion

Although many studies have reported plant activity against the nematode *H*. *contortus*, this activity is usually related to secondary metabolic compounds [11,12]. In fact, *S*. *anthelmia* has known anthelmintic activity [9], including against *H*. *contortus* [10]. However, this study is the first to provide evidence of *S*. *anthelmia* protein activity against *H*. *contortus*, opening a new field of research involving plant protein molecules with potential use against this parasite.

The leaf, stem and root fractions of *S*. *anthelmia* presented total protein contents of 51.71, 9.19 and 8.28 mg, respectively, and the variations are attributed to differences in the protein composition in each part of the plant. As plant protein content is affected by climatic and geographic variations and by nutrient availability in water and soil [30], the higher protein concentration in the leaves can be related to greater exposure to biotic and abiotic stresses in the environment [30,31].

The relationship between the exclusive proteins found in each fraction and anthelmintic activity is unclear because it appears that the same main classes of proteins are present but in different isoforms or subclasses. Possible synergism between or different concentrations of active proteins could be responsible for such differences in the anthelmintic activity of each fraction.

Proteins in *S*. *anthelmia* fractions were identified by MS. Proteases, chitinase, ATP sintase, fructokinase, ribosomal protein, glutathione reductase, and calmodulin were identified among all protein fractions.

Proteolytic enzymes are involved in a wide variety of functions and physiological processes, including hydrolysis of essential digestive proteins of herbivorous insects, phytonematoids and pathogenic microorganisms [32]. In our study, protease activity was detected in LPF, SPF and RPF (Table 1). Proteases act on the cuticle of the parasite, causing severe damage and resulting in nematode death [33,34].

Moreover, the nematode cuticle is a complex structure comprised primarily of proteins, carbohydrates and lipids, with collagen as the primary component [35,36]. Loss of the sheath by the third stage in larvae occurs in response to stimuli such as pH, temperature and the presence of carbon dioxide in the environment, inducing secretion of enzymes that promote digestion of the sheath and larval release [37,38,39]. Thus, the action of *S*. *anthelmia* fractions against larvae can be associated with the presence of proteases that hydrolyze important proteins that are necessary for migration processes. We obtained EC_50_ values of 0.11 to 0.21 mg mL^-1^ for LMIA and 0.47 to 0.78 mg mL^-1^ for LEIA (Table 2).

Because nematode proteases degrade the egg membrane during egg hatching [35], protease inhibitors may be associated with total or partial inhibition of proteolytic enzymes that are essential for development of the parasite. Interestingly, although protease inhibitor activity was detected in all fractions (Table 1), such a protein identified by mass spectrometry was found only in SPF. Some proteins are known to be bifunctional because they have multiple domains with different functions [39,40]. Bifunctional proteins present in the *S*. *anthelmia* fractions may have influenced the detection of protease inhibitor activity in LPF and SPF. In addition, uncharacterized proteins after proteome analysis may also have influenced the results (S1 Fig).

Chitinases were also identified in the fractions but at low activity (Table 1). These enzymes degrade chitin, an important component of the nematode egg shell [41,42]. In nematodes, chitinase degrades the chitin present in the cuticles of the egg [35] and larva, inhibiting their development or leading to death [43].

Another class of proteins involved in plant defense is ribonuclease (RNAse), a protein involved in the processing and degradation of RNA. Although its mechanisms are not known, RNAse participates in complex plant defense responses to pathogens, primarily against pathogenic fungi [44,45].

Lectin was not identified in the samples by mass spectrometry or *in vitro* assays (Table 1). The presence of lectins in plants has been associated with a protective role by interacting with the sensory structures of a parasite, lectins hinder root penetration by phytonematoids, reducing their mobility [46].

Differences in the structure of the egg membrane and the cuticle of infective nematode larvae may interfere with the anthelmintic activity of a product [35]. For example, a methanolic extract of *Morchella esculenta* acted on larval development but had no effect on eggs, larval migration or adult motility [47]. In addition, differences in the cuticles of *H*. *contortus* eggs and larvae as well as the types of proteins present can alter the mechanism of action of *Leucaena leucocephala* protein extracts [48]. Protein fractions from *S*. *anthelmia* exerted an effect during more than one stage of the life cycle of *H*. *contortus* (Table 2). This observation is relevant because the study of the cuticular composition of the egg membrane and nematode larvae is important for understanding the penetration of antiparasitic substances.

*In vivo* studies using ethanolic and aqueous extracts of *S*. *anthelmia* against gastrointestinal nematodes *Strongyloides* spp., *Oesophagostomum* spp., *Trichuris* spp., *Haemonchus* spp., and *Trichostrongylus* spp. of sheep have revealed reductions in fecal eggs and the survival of third-stage larvae [10]. In the present study, we show that the protein fractions obtained from *S*. *anthelmia*, which were free of secondary metabolism compounds, present anthelmintic effects toward *H*. *contortus* eggs and larvae. The approach we used to obtain the protein fractions is inexpensive, fast, comprehensive and easily reproducible. Additional studies should be performed to understand the role of plant bioactive proteins against *H*. *contortus* as well as to identify the likely mechanisms of action of these proteins. This information will contribute to the discovery of new drugs that are efficient *in vivo* against the nematode *H*. *contortus*.

## Supporting information

S1 Fig Total list of proteins identified by LC-ESI-MS/MS. (XLSX)

S2 Fig Venn diagram comparing proteins of the *S*. *anthelmia* fractions: LPF (leaf protein fraction); SPF (stem protein fraction) and RPF (root protein fraction). (DOCX)

S3 Fig Efficiency of *Spigelia anthelmia* fractions against *Haemonchus contortus* eggs and larvae. LPF: leaf protein fraction; SPF: stem protein fraction; RPF: root protein fraction. EHA: egg hatch assay; LEIA: larval exsheathment inhibition assay; LMIA: larval migration inhibition assay.
(DOCX)

S4 Fig Effect of the leaf protein fraction (A), stem protein fraction (B) and root protein fraction (C) of *Spigelia anthelmia* on *in vitro* exsheathment inhibition of *Haemonchus contortus* third-stage larvae.
(DOCX)
