# Supplementary figures and images for: In vitro anthelmintic effects of Spigelia anthelmia protein fractions against Haemonchus contortus
Source: PLoS One. 2017 Dec 15;12(12):e0189803. doi: 10.1371/journal.pone.0189803 (PMC5731696; doi:10.1371/journal.pone.0189803)

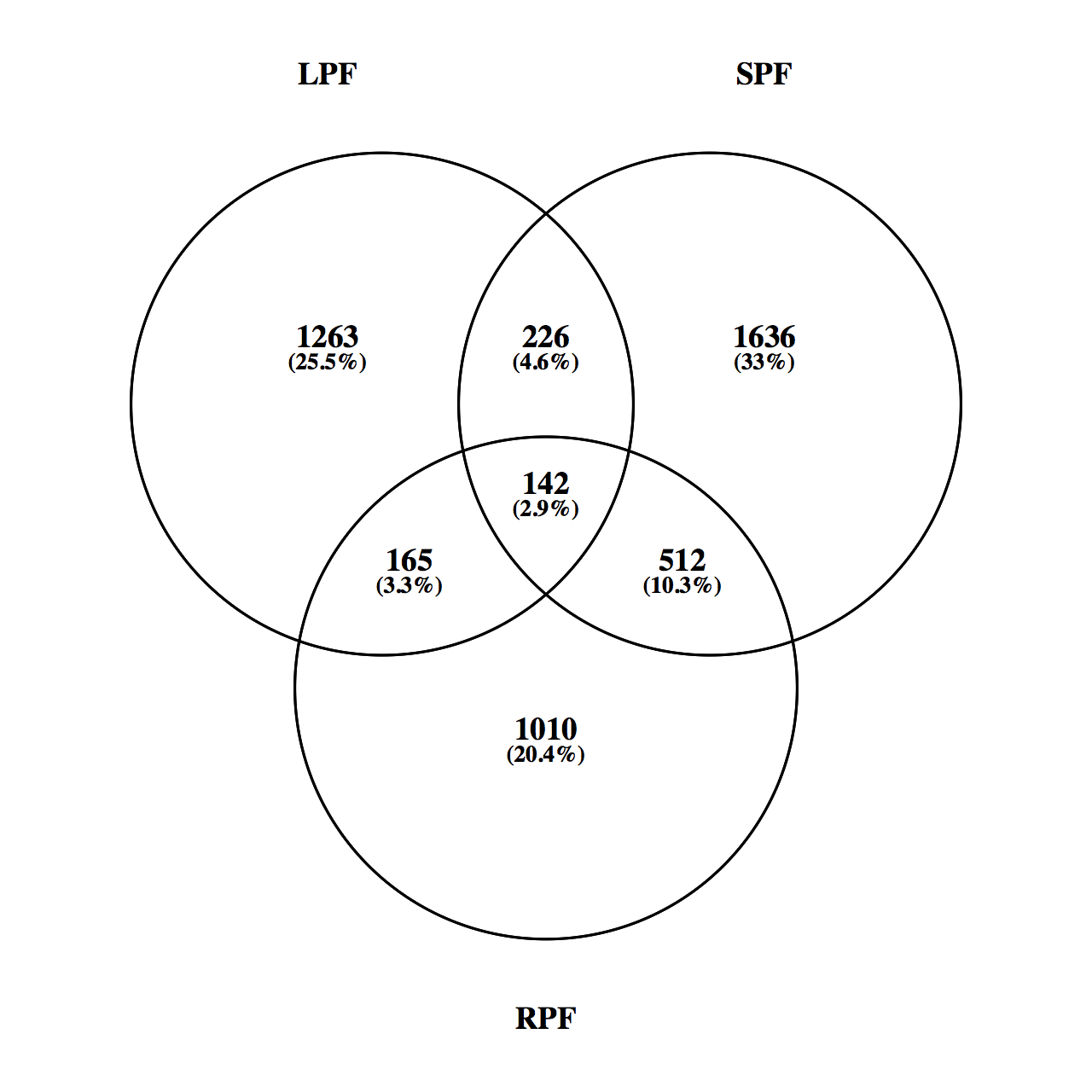

Supplement: S2 Fig — (DOCX) [file pone.0189803.s002.docx]

**Supporting information**

**4S Fig.**


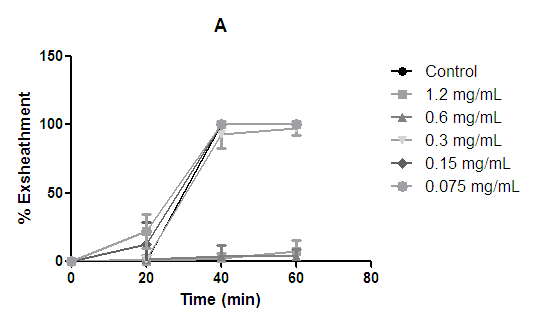

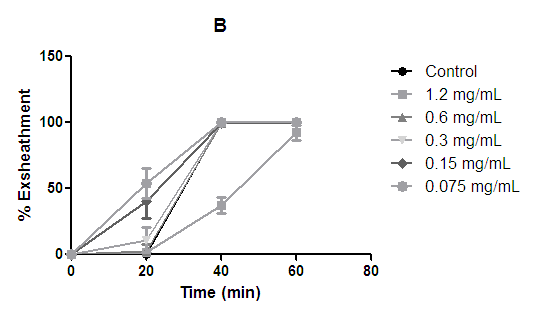

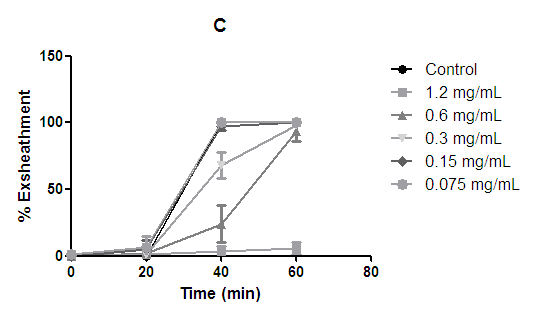


**A)**

**B)**

**C)**

Supplement: S4 Fig — Effect of the leaf protein fraction (A), stem protein fraction (B) and root protein fraction (C) of Spigelia anthelmia on in vitro exsheathment inhibition of Haemonchus contortus third-stage larvae. (DOCX) [file pone.0189803.s004.docx]
