# Supplementary material for: In vitro anthelmintic effects of Spigelia anthelmia protein fractions against Haemonchus contortus
Source: PLoS One. 2017 Dec 15;12(12):e0189803. doi: 10.1371/journal.pone.0189803 (PMC5731696; doi:10.1371/journal.pone.0189803)
Supplement: S3 Fig — LPF: leaf protein fraction; SPF: stem protein fraction; RPF: root protein fraction. EHA: egg hatch assay; LEIA: larval exsheathment inhibition assay; LMIA: larval migration inhibition assay. (DOCX) [file pone.0189803.s003.docx]

Supporting information

3S Fig.

| **EHA** | | | |
| --- | --- | --- | --- |
| **[ ] mg protein. mL^-1^** | **LPF** | **SPF** | **RPF** |
| Control | 0,00 | 0,00 | 0,00 |
| 2 | 100,00 ± 0,00 | 100,00 ± 0,00 | 100,00 ± 0,00 |
| 1 | 97,12 ± 1,74 | 93,68 ± 7,65 | 69,52 ± 9,80 |
| 0,5 | 94,79 ± 2,25 | 19,33 ± 4,26 | 14,40 ± 3,91 |
| 0,25 | 89,71 ± 5,04 | 12,60 ± 5,06 | 7,38 ± 2,09 |
| 0,125 | 11,12 ± 3,93 | 8,55 ± 2,07 | 2,55 ± 3,18 |
| 0,062 | 0,00 | 3,69 ± 2,93 | 0,00 |
| **LEIA** | | | |
| 1,2 | 91,36 ± 10,04 | 7,90 ± 5,59 | 96,94 ± 3,75 |
| 0,6 | 76,25 ± 47,50 | 0,00 | 10,63 ± 7,09 |
| 0,3 | 8,59 ± 3,09 | 0,00 | 2,17 ± 2,69 |
| 0,15 | 0,00 | 0,00 | 0,00 |
| 0,075 | 0,00 | 0,00 | 0,00 |
| **LMIA** | | | |
| 1 | 96,29 ± 2,45 | 82,01 ± 11,72 | 81,70 ± 7,73 |
| 0,5 | 83,27 ± 17,97 | 69,83 ± 6,31 | 70,59 ± 10,55 |
| 0,25 | 50,16 ± 3,01 | 68,52 ± 8,38 | 55,84 ± 6,81 |
| 0,125 | 14,72 ± 14,63 | 26,60 ± 3,12 | 46,00 ± 7,40 |
| 0,062 | 0,00 | 22,04 ± 10,56 | 33,89 ± 13,61 |
| 0,031 | 0,00 | 0,48 ± 7,93 | 0,43 ± 11,09 |
